# A Quantitative CT-Based Analysis of Vertebral Rotational Asymmetry and Pulmonary Function in Scoliosis

**DOI:** 10.3390/jcm15114154

**Published:** 2026-05-28

**Authors:** Beom-Su Kim, Ihnseok Chae, Jeuk Lee, Bong-Soon Chang, Sam Yeol Chang, Dong-Gune Chang, Hyoungmin Kim

**Affiliations:** 1 Department of Orthopedic Surgery, Seoul National University Hospital, 101, Daehak-ro, Jongno-gu, Seoul 03080, Republic of Korea; kbumsu2@gmail.com (B.-S.K.); bschang@snu.ac.kr (B.-S.C.);; 2 Department of Orthopedic Surgery, Inje University Sanggye Paik Hospital, 1342, Dongil-ro, Nowon-gu, Seoul 01757, Republic of Korea; ihnseokchae@gmail.com (I.C.); dgchangmd@gmail.com (D.-G.C.); 3 Department of Orthopedic Surgery, Inha University Hospital, 27, Inhang-ro, Jung-gu, Incheon 22332, Republic of Korea; jeuklee2@gmail.com

**Keywords:** scoliosis, computed tomography, vertebral rotation, axial rotation, pulmonary function, FVC, FEV1, rotational asymmetry

## Abstract

**Background/Objectives:** Scoliosis is a three-dimensional deformity involving coronal curvature, axial rotation, and sagittal imbalance, which may alter thoracic geometry and reduce ventilatory capacity. Traditional two-dimensional radiographic measures incompletely represent the complexity of axial rotation, and the apical vertebra is not always the most rotated vertebra. We aimed to determine whether computed tomography (CT)-derived three-dimensional vertebral rotation indices, particularly global rotational asymmetry between main and compensatory curves, are associated with pulmonary function impairment in a large heterogeneous scoliosis cohort. **Methods:** We retrospectively reviewed 250 patients with scoliosis who underwent full-spine CT and spirometry within a 1-year interval (2013–2023). Vertebral rotation was measured using the Aaro–Dahlborn method. Rotation indices included apical rotation (R(Apex)), averaged apical rotation across the apical vertebra and adjacent levels (R(Avg)), and rotational asymmetry defined as the absolute difference between rotation in the main and compensatory curves (ΔR(M–C)). Pulmonary function outcomes were FVC (L), FEV1 (L), FVC% and FEV1%. Pearson correlation and multivariate linear regression, adjusted for age, sex, height, and weight, were performed; sensitivity analyses, additionally adjusted for upright Cobb angle and thoracic kyphosis (TK) to evaluate whether ΔR(M–C) provided independent explanatory information, and subgroup analyses by etiology were performed. **Results:** The cohort had a mean age of 15.6 ± 5.7 years; 49.6% were female. All rotation indices showed significant negative correlations with pulmonary function in the overall cohort. ΔR(M–C) showed the strongest associations with FVC% (r = −0.66) and FEV1% (r = −0.64), as well as with absolute volumes (FVC, r = −0.59; FEV1, r = −0.58). In adjusted multivariate analyses, models incorporating ΔR(M–C) consistently demonstrated the highest explanatory performance compared with models based on R(Apex) or R(Avg). Subgroup analysis revealed the strongest associations in neurofibromatosis-related scoliosis (r = −0.87) and congenital scoliosis (r = −0.71). Associations were attenuated in neuromuscular subtypes and did not reach statistical significance in SMA. In sensitivity analyses adjusting for Cobb angle and thoracic kyphosis, ΔR(M–C) retained a robust independent association with FVC% (unstandardized B = −0.82 percentage points per 1°, 95% CI −0.98 to −0.66; *p* < 0.001; partial F = 103, *p* < 0.001), while neither Cobb angle nor TK remained statistically significant after adjustment for ΔR(M–C); comparable patterns were observed across FEV1%, FVC, and FEV1. **Conclusions:** CT-derived global rotational asymmetry between the main and compensatory curves is strongly associated with pulmonary function impairment in scoliosis and demonstrates superior explanatory performance to single-level rotation indices and retains independent explanatory value after adjustment for conventional 2D radiographic parameters (Cobb angle and thoracic kyphosis). Incorporating a CT-derived metric may complement traditional two-dimensional assessments for functional risk stratification.

## 1. Introduction

Scoliosis is classically defined as a structural lateral curvature of the spine, but clinically relevant scoliosis is inherently a three-dimensional deformity involving coronal curvature, axial rotation, and sagittal profile alterations [1,2,3]. Axial rotation contributes to rib cage torsion and asymmetric rib excursion, with consequent alterations in chest wall mechanics that may increase the torsional stiffness of the thoracic cage and reduce chest wall compliance. These changes can restrict lung expansion and reduce ventilatory capacity, particularly in thoracic curves and severe deformity [4,5]. Pulmonary impairment in scoliosis is typically restrictive, reflected by reductions in forced vital capacity (FVC) and forced expiratory volume in one second (FEV1), and has long been recognized as an important determinant of morbidity and perioperative risk in select patient populations [4,6,7].

Despite recognition of a structural–functional relationship, quantifying which spinal deformity features best explain pulmonary compromise remains challenging. Historically, most clinical investigations and decision-making have relied on two-dimensional radiographic measures such as Cobb angle, thoracic kyphosis, or simple apical vertebral rotation measured on radiographs [7,8]. However, the Cobb angle does not directly quantify axial rotation, and single-level rotation metrics may miss global rotational imbalance. Importantly, the “apical vertebra” of a coronal curve is not necessarily the vertebra with maximal axial rotation, and thoracic deformation is influenced by the distribution of rotation across multiple levels rather than a single segment [2,5,9,10]. Accordingly, single-level rotation metrics may be limited because thoracic deformity reflects distributed multi-level rotation and the interaction between structural and compensatory curves. This raises the possibility that global measures of rotational asymmetry, particularly the imbalance between main and compensatory curves, may better capture thoracic distortion and its functional consequences. Recent studies incorporating multiple radiographic deformity parameters have similarly reported that Cobb angle alone correlates only modestly with pulmonary function (r approximately −0.38 to −0.40), and that two-dimensional thoracic kyphosis correlates only weakly with spirometric outcomes (r approximately 0.23–0.25), suggesting that conventional 2D measurements capture only a fraction of the three-dimensional thoracic deformity relevant to ventilatory restriction [11].

Computed tomography (CT) permits high-resolution three-dimensional assessment of vertebral rotation and has been used to define rotation indices and thoracic deformity parameters in scoliosis [1,5,10]. The Aaro–Dahlborn technique, based on the relationship between the spinal canal midline and axial imaging plane, is a well-established method for quantifying vertebral rotation [1]. However, prior work correlating CT-derived axial rotation with pulmonary function is limited, often focused on idiopathic scoliosis, relatively small cohorts, or single-rotation parameters instead of comprehensive indices capturing global rotational balance [5,9,10]. Additionally, scoliosis is etiologically heterogeneous; congenital, neuromuscular, and syndromic disorders may influence lung function through different mechanisms (thoracic rigidity, muscular weakness, intrinsic pulmonary disease), underscoring the need for generalizable quantitative predictors. Accordingly, while CT is not advocated for routine screening, it is frequently obtained in complex or high-risk scoliosis phenotypes and for surgical planning, providing an opportunity to derive functionally relevant 3D biomarkers from imaging already acquired. Other three-dimensional indices, such as rotation-corrected thoracic kyphosis, have likewise been proposed as independent predictors of pulmonary function in adolescent idiopathic scoliosis, reinforcing the view that three-dimensional assessment captures functionally relevant deformity components not fully represented on plain radiographs [12].

The primary objective of this study was to evaluate, in a large and etiologically diverse cohort, the association between CT-derived vertebral rotation indices and pulmonary function. We specifically tested the hypothesis that global rotational asymmetry between the main and compensatory curves, quantified as ΔR(M–C), would demonstrate stronger association and explanatory power for pulmonary function outcomes than single-level apical rotation R(Apex) or averaged apical rotation R(Avg). Secondary aims included subgroup comparisons across etiologic categories and provision of practical, clinically interpretable rotation–function relationships that could enhance preoperative functional risk assessment, with an emphasis on clinically interpretable indices that capture global torsional imbalance. As a prespecified sensitivity analysis, we additionally evaluated whether ΔR(M–C) retained independent explanatory value after adjustment for conventional radiographic deformity parameters—namely, Cobb angle and thoracic kyphosis—which are the most widely used 2D measures of scoliotic deformity in clinical practice.

## 2. Materials and Methods

### 2.1. Study Design and Population

This was a retrospective cross-sectional observational study. Consecutive patients with scoliosis who underwent (1) full-spine CT and (2) standard pulmonary function testing (spirometry) within a 1-year interval between January 2013 and December 2023 were considered eligible. Inclusion criteria were: (i) diagnosis of scoliosis, (ii) availability of a CT suitable for vertebral rotation measurement, and (iii) spirometry results including FVC and FEV1 values with percent-predicted indices. Exclusion criteria were: (i) missing key covariates (age, sex, height, weight), (ii) inadequate CT quality for rotation measurement at target vertebral levels, or (iii) non-interpretable spirometry.

A total of 250 patients satisfied the criteria and comprised the analytic cohort. Patients were categorized into the following diagnostic groups: adolescent idiopathic scoliosis (AIS), congenital scoliosis, neuromuscular scoliosis, and syndromic scoliosis. For subgroup correlation analysis (Table 1), etiologic subgroups were further separated as AIS, CMD, congenital scoliosis, DMD, NF, other neuromuscular, SMA, and other syndromic disorders (*n* values below). The inclusion of multiple etiologic subtypes was intentional, reflecting real-world scoliosis populations and enabling evaluation of a rotation-based metric across diverse phenotypes, with planned subgroup analyses to assess whether the strength of association and explanatory performance differ by etiology.

### 2.2. CT Acquisition and Vertebral Rotation Measurement

All CT examinations were obtained in the supine position following institutional protocols using multidetector CT scanners. Standard spine imaging parameters were applied, including a slice thickness of 1–3 mm, tube voltage of 100–120 kVp, automatic tube current modulation, and reconstruction with a standard soft-tissue kernel; axial source images were used for rotation analysis. All images were retrieved from the institutional Picture Archiving and Communication System (INFINITT Healthcare, Seoul, Republic of Korea) and reformatted along the anatomic axes when necessary. Vertebral rotation was quantified on axial CT slices using the Aaro–Dahlborn method [1]. Briefly, rotation is defined as the angle between the mid-sagittal line of the spinal canal and the axial imaging plane. The axial slice for each vertebra was selected at the geometric center of the vertebral body, and measurements were performed at each vertebral level relevant to the main and compensatory curves, enabling the derivation of rotation indices described below. Measurements were performed by a single trained observer (an orthopedic spine fellow) blinded to spirometry results; to assess measurement reliability, a 10% random subsample was independently re-measured and intraclass correlation coefficients (ICCs) were computed, with values in the “excellent” range (ICC > 0.90) accepted as the threshold for analytic inclusion. Maximum rotation of the main curve (RM) was defined as positive, while maximum rotation of the compensatory curve (RC) was defined in the opposite direction (negative). Rotational asymmetry, ΔR(M–C), was calculated as the absolute difference between RM and RC, thereby reflecting the combined magnitude of rotation across oppositely directed curves.

Rotation indices (predictors)

We used three primary CT-derived rotation indices for the core analyses:

R(Apex): Axial rotation angle of the apical vertebra of the main structural curve.

R(Avg): Mean axial rotation of the apical vertebra and the immediately adjacent cranial and caudal levels (three-vertebra average).

ΔR(M–C): Rotational asymmetry, defined as the absolute difference between RM (maximum rotation in the main curve) and RC (most counter-rotated segment in the compensatory curve, i.e., maximal rotation opposite the main curve direction) (i.e., ΔR(M–C) = |RM − RC|, which approximates |RM| + |RC| when rotations are opposite in sign). This parameter was intended to quantify global rotational imbalance across the thoracic spine.

Pulmonary function testing (outcomes)

All patients underwent standard pulmonary function testing in the seated position by trained respiratory technicians, performed in accordance with the joint American Thoracic Society/European Respiratory Society (ATS/ERS) standardization recommendations [13]. Predicted values for FVC and FEV1 were derived using the Global Lung Function Initiative (GLI) 2012 reference equations [14], with appropriate adjustments for age, sex, height, and ethnicity. The primary pulmonary outcomes were:

FVC (L) and FVC%

FEV1 (L) and FEV1%

Given the predominantly restrictive physiology observed in scoliosis, FVC and FVC% were considered primary outcomes [4,6,7].

### 2.3. Conventional Radiographic Parameters

To enable comparison with established two-dimensional measures of spinal deformity, the coronal Cobb angle and thoracic kyphosis (TK) were additionally measured for each patient. The coronal Cobb angle of the main structural curve was measured both on supine reformatted CT images (Cobb_CT) and on upright (sitting or standing) full-spine posteroanterior radiographs (Cobb_XR) using the standard end-vertebra technique [15]. Thoracic kyphosis was measured between the superior endplate of T5 and the inferior endplate of T12 on the corresponding sagittal CT reconstructions (TK_CT) and lateral upright radiographs (TK_XR). All measurements were performed in the PACS environment (INFINITT Healthcare, Seoul, Republic of Korea) by the same trained observer described above, blinded to the spirometry results. These conventional radiographic parameters were used in sensitivity analyses to evaluate whether ΔR(M–C) retained independent explanatory value after adjustment for established two-dimensional indices of deformity severity.

### 2.4. Covariates

Multivariate models were adjusted for age, sex, height, and weight, selected a priori due to their known influence on lung volumes and percent-predicted computations.

### 2.5. Statistical Analyses

Continuous variables were summarized as mean ± standard deviation. Pearson correlation coefficients (r) were calculated to assess linear associations between rotation indices and pulmonary outcomes. Multivariate linear regression was performed for each pulmonary outcome with each rotation index (separate models) adjusted for age, sex, height, and weight. Model performance was assessed using adjusted R^2^. Subgroup analyses were performed using Pearson correlation between ΔR(M–C) and pulmonary function parameters (FVC, FVC%, FEV1, and FEV1%) by etiologic subgroup.

Statistical significance was set at *p* < 0.05, with emphasis on effect sizes and consistency across outcomes rather than sole reliance on *p*-values in accordance with reporting recommendations for observational studies.

Distributional assumptions for continuous variables were examined using the Shapiro–Wilk test and visual inspection of histograms and Q–Q plots, and parametric methods were applied when assumptions were satisfied. For multivariate linear regression models, multicollinearity was assessed using the variance inflation factor (VIF), with all predictors in adjusted models confirmed to have VIF values below 5. Incremental explanatory value of each rotation index was evaluated by computing the change in adjusted R^2^ (ΔR^2^) when the index was added to base anthropometric models containing age, sex, height, and weight. All analyses were performed using R version 4.3.0 (R Foundation for Statistical Computing, Vienna, Austria), with two-sided *p*-values reported and statistical significance set at *p* < 0.05.

To address the contribution of conventional radiographic parameters, sensitivity analyses were performed in which Cobb angle (measured on upright radiographs, Cobb_XR) and thoracic kyphosis (TK_XR) were added as additional covariates in multivariate regression models. The independent contribution of ΔR(M–C) was evaluated by (i) comparing nested models with and without ΔR(M–C) using both the change in adjusted R^2^ (ΔR^2^) and a partial F-test of the corresponding likelihood-ratio comparison; (ii) reporting both unstandardized (B; change in outcome per 1° increase in the predictor) and standardized regression coefficients (β; change in outcome SD per 1 SD increase in the predictor) for ΔR(M–C) in the full model, together with 95% confidence intervals and two-sided *p*-values; and (iii) repeating the sensitivity analysis across all four spirometric outcomes (FVC, FVC%, FEV1, and FEV1%) to evaluate robustness. Multicollinearity within the full model was again confirmed by ensuring all variance inflation factors remained below 5.

### 2.6. Ethical Approval

This study was approved by the Institutional Review Board of Seoul National University Hospital (IRB No. H-2402-077-1512, approved on 13 March 2024). Informed consent was waived due to the retrospective nature of the study.

## 3. Results

### 3.1. Patient Characteristics

The study cohort included 250 patients with scoliosis (mean age, 15.6 ± 5.7 years; 49.6% female). Major diagnostic categories were AIS (*n* = 57), congenital scoliosis (*n* = 24), neuromuscular scoliosis (*n* = 106), and syndromic scoliosis (*n* = 63). Overall spirometry demonstrated predominantly restrictive ventilatory patterns, with a mean FVC of 2.03 ± 1.04 L and a mean FEV1 of 1.77 ± 0.92 L.

### 3.2. Correlation Between Rotation Indices and Pulmonary Function (Table 2)

Pearson correlation analyses demonstrated significant inverse associations between all CT-derived rotation indices and pulmonary function outcomes (all *p* values < 0.001) (Table 2). ΔR(M–C) consistently showed the strongest negative correlations across all pulmonary indices, with the highest magnitude observed for percent-predicted outcomes: FVC% (r = −0.66) and FEV1% (r = −0.64). ΔR(M–C) also correlated strongly with absolute volumes: FVC (r = −0.59) and FEV1 (r = −0.58).

**Table 2:** Pearson correlation coefficients between vertebral rotation indices and pulmonary function.

Outcome Variable	R(Apex)	R(Avg)	ΔR(M–C)
FEV1 (L)	−0.46	−0.47	−0.58
FEV1%	−0.50	−0.52	−0.64
FVC (L)	−0.47	−0.48	−0.59
FVC%	−0.53	−0.54	−0.66

All correlations were statistically significant (*p* < 0.001).

By comparison, R(Apex) and R(Avg) showed more moderate correlations with pulmonary outcomes, including FVC% (r = −0.53 and −0.54), FEV1% (r = −0.50 and −0.52), and absolute volume measures (r approximately −0.47 to −0.48). Across all outcomes, R(Avg) was slightly stronger than R(Apex), but differences were small.

**Figure 1:**
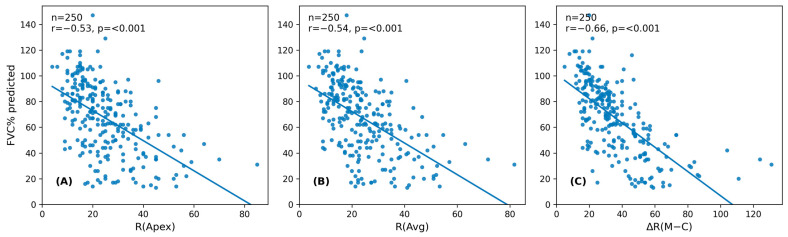
Scatter plots illustrating the relationships between CT-based vertebral rotation indices and forced vital capacity percent predicted (FVC%) in the overall cohort (*n* = 250). (**A**) Apical vertebral rotation (R(Apex)), (**B**) averaged apical rotation including adjacent levels (R(Avg)), and (**C**) rotational asymmetry between the main and compensatory curves (ΔR(M–C)). Solid lines represent linear regression fits. ΔR(M–C) demonstrates the strongest linear association with FVC%, with reduced dispersion and greater explanatory strength compared with single-level rotation metrics.

**Figure 2:**
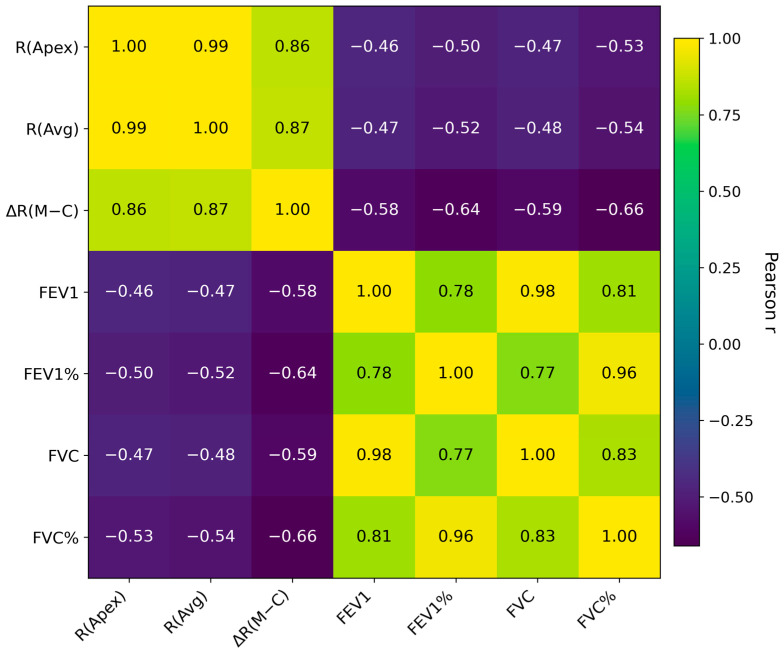
Heatmap showing Pearson correlation coefficients between vertebral rotation indices (R(Apex), R(Avg), and ΔR(M–C)) and pulmonary function parameters (FEV1, FEV1%, FVC, and FVC%). Color intensity reflects the magnitude and direction of correlation (range −1 to +1). ΔR(M–C) consistently shows the strongest negative correlations across all pulmonary function outcomes.

Overall, these correlation patterns demonstrated a consistent hierarchy of association strength: ΔR(M–C) > R(Avg) ≈ R(Apex), indicating that global rotational asymmetry shows stronger associations with pulmonary impairment than localized apical rotation measures. Scatter plots of individual rotation indices against FVC% in the overall cohort are shown in Figure 1, and the full correlation matrix across all rotation indices and pulmonary function parameters is summarized in Figure 2.

### 3.3. Multivariate Regression Adjusted for Age, Sex, Height, and Weight (Table 3)

In multivariate linear regression models adjusted for age, sex, height, and weight, each rotation index remained a significant independent predictor of pulmonary outcomes (Table 3). Model explanatory performance differed among indices. For FEV1%, the adjusted R^2^ was 0.36 for R(Apex), 0.37 for R(Avg), and 0.47 for ΔR(M–C). For FVC%, adjusted R^2^ values were 0.40 for R(Apex), 0.41 for R(Avg), and 0.51 for ΔR(M–C).

**Table 3:** Adjusted R^2^ values for individual vertebral rotation indices after adjustment for age, sex, height, and weight.

Outcome Variable	R(Apex)	R(Avg)	ΔR(M–C)
FEV1 (L)	0.59	0.60	0.65
FEV1%	0.36	0.37	0.47
FVC (L)	0.60	0.61	0.66
FVC%	0.40	0.41	0.51

Across percent-predicted outcomes, models incorporating ΔR(M–C) consistently demonstrated the highest explanatory performance, exceeding those incorporating R(Apex) or R(Avg). For absolute volume outcomes, similar patterns were observed. Across all outcomes, R(Avg) yielded modestly higher adjusted R^2^ than R(Apex), consistent with the concept that multi-level apical averaging better represents regional deformity than a single apical measurement.

**Figure 3:**
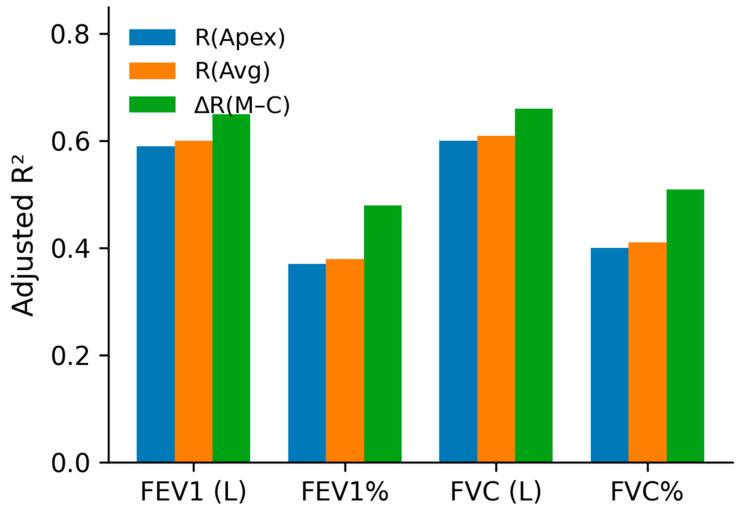
Comparison of adjusted R^2^ values from multivariate linear regression models predicting pulmonary function parameters using individual rotation indices, adjusted for age, sex, height, and weight. Rotational asymmetry (ΔR(M–C)) demonstrates the highest explanatory power across all outcomes, outperforming R(Apex) and R(Avg).

Because absolute spirometric volumes are strongly influenced by anthropometrics, we additionally quantified the incremental explanatory contribution of each rotation index beyond covariates alone (Δadjusted R^2^). ΔR(M–C) yielded the largest incremental gains across all outcomes, with particularly pronounced improvements for percent-predicted indices (Δadjusted R^2^ = 0.308 for FVC% and 0.298 for FEV1%; Supplementary Table S3). A comparison of adjusted R^2^ values across rotation indices for each pulmonary function outcome is shown in Figure 3. 

### 3.4. Etiologic Subgroup Analysis for ΔR(M–C) Versus FVC% (Table 1)

Subgroup analysis demonstrated that the strength of association between rotational asymmetry and pulmonary impairment varied by etiology. The strongest correlation between ΔR(M–C) and FVC% was observed in neurofibromatosis-related scoliosis (NF) (*n* = 18; r = −0.87), followed by congenital scoliosis (*n* = 24; r = −0.71). AIS showed a moderate correlation (*n* = 57; r = −0.52), while neuromuscular subgroups including SMA (*n* = 20; r = −0.36) and DMD (*n* = 34; r = −0.41) demonstrated weaker associations. CMD (*n* = 22; r = −0.61) showed a relatively strong association among neuromuscular etiologies. Other syndromic disorders (*n* = 45) had r = −0.40, while other neuromuscular subtypes (*n* = 30) had r = −0.46.

Collectively, subgroup analyses demonstrated a stronger association between rotational asymmetry and pulmonary impairment in structurally rigid phenotypes (e.g., NF-related and congenital scoliosis), with attenuated associations observed in neuromuscular subtypes in which respiratory muscle weakness may contribute substantially to pulmonary impairment [4] (Table 1).

### 3.5. Sensitivity Analysis Incorporating Conventional Radiographic Parameters (Table 4)

To address whether ΔR(M–C) provided explanatory information independent of conventional 2D radiographic deformity parameters, sensitivity analyses were performed in which Cobb angle (measured on standing/sitting radiographs; Cobb_XR) and T5–T12 thoracic kyphosis (TK_XR) were incrementally entered into multivariate models predicting FVC%. The base model adjusted for age, sex, height, and weight (adjusted R^2^ = 0.201). Addition of Cobb_XR alone increased the adjusted R^2^ to 0.299 (ΔR^2^ = +0.098), and TK_XR alone produced only a marginal increment (adjusted R^2^ = 0.220; ΔR^2^ = +0.019). A model containing both Cobb_XR and TK_XR together with anthropometric covariates explained 29.7% of FVC% variance. In contrast, addition of ΔR(M–C) to the base model alone yielded an adjusted R^2^ of 0.509 (ΔR^2^ = +0.308), and a full model containing all three deformity parameters (Cobb_XR, TK_XR, and ΔR(M–C)) produced an adjusted R^2^ of 0.505 (Table 4).

**Table 4:** Sensitivity analysis: incremental explanatory performance of CT-derived rotational asymmetry, ΔR(M–C), relative to conventional 2D radiographic deformity parameters (Cobb angle and thoracic kyphosis) for prediction of FVC%. All models adjusted for age, sex, height, and weight. B values are unstandardized regression coefficients (change in FVC% per 1° increase in the predictor); standardized β coefficients are reported separately for the full model in the text.

Model (Covariates: Age, Sex, Height, Weight)	Adjusted R^2^	ΔR^2^ vs. Base	B for ΔR(M–C)	*p* for ΔR(M–C)
Base (anthropometrics only)	0.201	—	—	—
Base + Cobb_XR	0.299	+0.098	—	—
Base + TK_XR	0.220	+0.019	—	—
Base + Cobb_XR + TK_XR	0.297	+0.096	—	—
Base + ΔR(M–C)	0.509	+0.308	−0.83	<0.001
Base + Cobb_XR + ΔR(M–C)	0.507	+0.306	−0.82	<0.001
Base + Cobb_XR + TK_XR + ΔR(M–C) (full)	0.505	+0.304	−0.82	<0.001

Cobb_XR = Cobb angle measured on upright (sitting/standing) radiographs. TK_XR = T5–T12 thoracic kyphosis measured on upright lateral radiographs. ΔR(M–C) = absolute rotational asymmetry between main and compensatory curves measured on axial CT, calculated as the absolute difference between maximum rotation of the main curve (RM, taken as positive) and maximum rotation of the compensatory curve (RC, taken in the opposite sign), with values reported as absolute magnitudes. B = unstandardized regression coefficient (change in FVC% per 1° increase in the predictor). All variance inflation factors in the full model were <2.1, indicating no problematic multicollinearity.

In the full multivariate model, ΔR(M–C) retained a strong and statistically significant independent association with FVC% (unstandardized B = −0.82 percentage points per 1° increase, 95% CI −0.98 to −0.66; standardized β = −0.57; *p* < 0.001), whereas Cobb_XR (B = −0.022, *p* = 0.778) and TK_XR (B = −0.010, *p* = 0.901) were not statistically significant after adjustment for ΔR(M–C). The incremental adjusted R^2^ obtained by adding ΔR(M–C) to a model already containing Cobb_XR and TK_XR was +0.208. All variance inflation factors in the full model were below 2.1, indicating that multicollinearity did not account for these findings. Comparable results were obtained when supine CT-derived Cobb_CT was substituted for Cobb_XR, although the explanatory contribution of Cobb_CT alone was smaller (adjusted R^2^ for base + Cobb_CT = 0.234; ΔR^2^ = +0.033), consistent with the known attenuation of coronal curve magnitude in the supine position.

## 4. Discussion

This study demonstrates that CT-derived vertebral rotation indices are strongly associated with pulmonary impairment in scoliosis, with global rotational asymmetry, ΔR(M–C), showing the highest and most consistent explanatory performance among the evaluated metrics. Across the full cohort, models incorporating ΔR(M–C) demonstrated stronger associations with both absolute and percent-predicted spirometric indices than models based on R(Apex) or R(Avg). In covariate-adjusted multivariate models, these models achieved the highest explanatory performance for FEV1 and FVC outcomes. Collectively, these findings support the concept that pulmonary restriction in scoliosis is not merely a function of localized deformity at an “apex,” but rather reflects the integrated three-dimensional imbalance of the thoracic spine and rib cage.

From a biomechanical perspective, a global measure of rotational imbalance may more accurately reflect the thoracic mechanical constraints that are associated with restrictive ventilatory physiology. Scoliosis-related pulmonary impairment has been attributed to reduced chest wall compliance, altered rib kinematics, and impaired symmetric expansion of the thoracic cage [4,6]. Accumulated axial rotation across multiple vertebral levels may be associated with increased torsional stiffness of the thoracic cage through coupled spine–rib mechanics, with asymmetric rib excursion and reduced chest wall compliance during respiration as potential contributing mechanisms. Such global torsional deformation is not fully captured by coronal curvature magnitude alone. Accordingly, while Cobb angle correlates with deformity severity, prior studies have suggested that pulmonary impairment cannot be inferred to a clinically useful degree from Cobb angle alone and that deformity–function relationships are multifactorial [7].

The ΔR(M–C) parameter was designed to quantify this integrated torsional deformation by capturing not only maximal rotation within the main curve but also the counter-rotational component present in the compensatory region. Functionally, this parameter approximates “how twisted the thorax is as a system,” rather than “how rotated one vertebra is.” An increased ΔR(M–C) reflects a state in which both the main and compensatory curves remain rotationally rigid even in the supine position, which may reflect reduced rotational flexibility across the thoracic spine and may be associated with a more globally constrained thoracic cage. This conceptual distinction may partly explain why ΔR(M–C) demonstrated reduced dispersion and stronger relationships to FVC% and FEV1% compared with R(Apex) and R(Avg). Consistent with prior conceptual frameworks emphasizing the thorax as a coupled spine–rib cage structure, global deformation indices have increasingly been advocated as more functionally relevant descriptors of scoliosis-related deformity [5,10].

Our findings align with both the classic and contemporary literature, demonstrating a structural–functional relationship between thoracic deformity and pulmonary function. Koumbourlis described scoliosis-associated reductions in chest wall compliance and secondary decreases in lung compliance, leading to increased work of breathing and, in severe cases, chronic respiratory failure [4]. Kearon and colleagues demonstrated that features of deformity are major determinants of impairment in idiopathic thoracic scoliosis and emphasized complexity beyond the Cobb angle alone [7]. Newton et al. similarly demonstrated associations between thoracic deformity and pulmonary function in a large AIS cohort [6]. The present study extends these observations by providing quantitative evidence that a CT-derived measure of global rotational asymmetry serves as a robust functional correlate across a large and etiologically diverse scoliosis population.

An additional observation in this study was that correlations were consistently stronger for percent-predicted pulmonary indices (FVC% and FEV1%) than for absolute lung volumes. Percent-predicted values normalize pulmonary function for age, sex, and body size and are commonly used to interpret restrictive impairment, particularly in pediatric and adolescent populations [4,7]. Stronger associations with percent-predicted outcomes suggest that rotational deformity is associated with pulmonary function beyond what would be expected from anthropometric variation alone. This interpretation is supported by our multivariate models, in which ΔR(M–C) remained the strongest explanatory variable even after adjustment for age, sex, height, and weight.

Subgroup analyses further demonstrated etiologic heterogeneity in the relationship between rotational asymmetry and pulmonary impairment. The strongest associations were observed in neurofibromatosis-related scoliosis (r = −0.87) and congenital scoliosis (r = −0.71), conditions characterized by substantial structural thoracic distortion and rigidity. In contrast, neuromuscular subtypes such as SMA and DMD exhibited weaker associations (r approximately −0.36 to −0.41), likely reflecting the dominant contribution of respiratory muscle weakness and impaired ventilatory mechanics in these conditions rather than a limitation of rotational metrics per se [4]. The relatively strong association observed in CMD (r = −0.61) highlights the heterogeneity within neuromuscular scoliosis and suggests that structural rigidity and deformity-driven restriction may remain important determinants of pulmonary function in select subgroups.

Incremental R^2^ analyses further demonstrated that the superiority of ΔR(M–C) was not merely driven by anthropometric effects but reflected a substantial independent contribution of global rotational asymmetry to pulmonary impairment.

Sensitivity analyses incorporating conventional 2D radiographic deformity parameters further reinforced the independent explanatory value of ΔR(M–C). When Cobb angle measured on upright radiographs (Cobb_XR) and T5–T12 thoracic kyphosis (TK_XR) were entered into multivariate models predicting FVC%, both contributed only modestly to model fit (adjusted R^2^ = 0.299 and 0.220, respectively), and a combined Cobb + TK model explained 29.7% of variance—closely comparable to Cobb alone. In contrast, adding ΔR(M–C) to the same anthropometric base model raised the adjusted R^2^ to 0.509, and ΔR(M–C) retained a robust independent association in the full model containing Cobb_XR, TK_XR, and anthropometrics (unstandardized B = −0.82 percentage points per 1° increase in ΔR(M–C); 95% CI −0.98 to −0.66; *p* < 0.001; standardized β = −0.57), while the contributions of Cobb_XR and TK_XR lost statistical significance after adjustment for ΔR(M–C). These findings are consistent with prior work showing that conventional 2D coronal and sagittal measurements explain only a fraction of pulmonary function variance in scoliosis. Farrell and Garrido reported similarly modest correlations between Cobb angle and pulmonary function (Cobb–FEV1 r = −0.40; Cobb–FVC r = −0.38) and weak correlations between 2D thoracic kyphosis and pulmonary function (r ≈ 0.23–0.25) in 170 preoperative thoracic AIS patients [11]. Ohashi et al. additionally demonstrated that three-dimensional thoracic kyphosis, corrected for vertebral rotation, was an independent predictor of pulmonary function (standardized β < 0.32), while plain 2D TK measurements were less informative [12]. The weak linear association between 2D TK and pulmonary function observed in our cohort (TK_XR vs. FVC% r = −0.16) is consistent with several mechanisms recognized in the literature. First, the relationship between TK and pulmonary function is non-linear and bidirectional: both hypokyphosis (loss of normal kyphosis) and hyperkyphosis are associated with reduced lung volumes through distinct mechanisms—hypokyphosis through tracheobronchial compression with documented lumen area reductions of up to 66% in the lower lobar bronchus [16], and hyperkyphosis through global thoracic distortion. In our cohort, hypokyphosis (<10°) was present in 13.6% and hyperkyphosis (>40°) in 13.2%, attenuating linear Pearson correlations. Second, TK and Cobb measurements obtained in the supine position systematically underestimate upright values (in our cohort, TK_CT averaged 16.0° versus TK_XR 24.6°), reflecting well-established postural effects on spinal alignment. Third, in the presence of substantial axial rotation, 2D lateral projection of the spine introduces measurement bias that overestimates apparent kyphosis [12]. The fact that ΔR(M–C) retained a robust independent explanatory value after simultaneous adjustment for Cobb_XR and TK_XR therefore supports the interpretation that global rotational asymmetry captures a functionally relevant dimension of thoracic deformity that is not adequately represented by conventional 2D measurements. Formal nested-model comparison confirmed this interpretation: adding ΔR(M–C) to a reduced model already containing Cobb_XR, TK_XR, and anthropometric covariates produced a highly significant improvement in model fit for FVC% (partial F = 103.0, *p* < 0.001; ΔAIC = −86.7), indicating substantial information gain rather than redundant variance. In clinical terms, the unstandardized coefficient implies that each 10° increase in ΔR(M–C) is associated with an approximately 8 percentage-point reduction in FVC%. Comparable patterns were observed for the remaining spirometric outcomes (FEV1%, FVC, FEV1), with ΔR(M–C) remaining the only significant deformity predictor in the full model in each case (all *p* < 0.001; partial F values 70.6–103.0 over the Cobb_XR + TK_XR + anthropometric reduced model), supporting the robustness of these findings across multiple spirometric endpoints.

These findings have important clinical implications. Preoperative assessment of pulmonary function is critical in scoliosis management, particularly when contemplating extensive thoracic fusion or corrective surgery in patients at risk for restrictive impairment [6]. However, spirometry is not always feasible in all settings or patient populations, and imaging-derived markers may offer an additional opportunity to contextualize associated functional risk when available. In this context, a CT-derived measure such as ΔR(M–C) may therefore provide a quantitative biomarker reflecting the global torsional burden of the thorax, particularly in patients undergoing CT for surgical planning or in complex scoliosis phenotypes.

Beyond risk contextualization at the cohort level, the present findings suggest several specific possible applications of ΔR(M–C) in clinical practice. First, in patients undergoing CT for preoperative surgical planning or evaluation of complex deformity, ΔR(M–C) could be incorporated alongside conventional radiographic parameters as part of an integrated, three-dimensional preoperative risk assessment, potentially identifying patients at elevated risk of ventilatory restriction who may benefit from preoperative respiratory optimization, anesthetic risk stratification, or postoperative pulmonary monitoring. Second, because ΔR(M–C) is derived directly from existing axial CT images and does not require additional imaging or specialized equipment, the index lends itself to automated or semi-automated derivation within existing radiology workflows, supporting scalable deployment without additional patient burden. Third, in patient populations in whom spirometry is technically difficult or unreliable—including young children, patients with cognitive or neuromuscular limitations, or those with severe deformity precluding standardized testing—ΔR(M–C) may provide a complementary, imaging-based indicator of associated functional burden when spirometry is not feasible. Fourth, the etiologic heterogeneity observed in subgroup analyses suggests that the clinical utility of ΔR(M–C) may differ across scoliosis phenotypes: the strongest associations were observed in structurally rigid conditions such as neurofibromatosis-related and congenital scoliosis, in which ΔR(M–C) may be particularly informative, whereas in neuromuscular subtypes—where respiratory muscle weakness is a dominant determinant of ventilatory capacity—imaging-based rotational metrics should be interpreted as a complement to, rather than substitute for, direct functional and respiratory muscle assessment. Finally, although prospective threshold values for clinical decision-making cannot be derived from the present cross-sectional data, the consistent dose–response pattern observed across spirometric endpoints suggests that future studies could explore whether specific ΔR(M–C) cut-points could be developed for risk stratification, triage of patients for prioritized PFT, or selection of candidates for more comprehensive three-dimensional thoracic assessment.

### 4.1. Limitations

Several limitations warrant consideration. First, the retrospective design introduces potential selection bias, as patients who underwent both full-spine CT and spirometry within a one-year interval likely represent a selected subset of the broader scoliosis population—typically those evaluated for surgical planning or with greater clinical concern for respiratory compromise. As a consequence, the cohort is likely enriched for more severe or rigid deformities, and the magnitude of associations observed here may not fully generalize to milder, community-based, or screening-detected scoliosis populations. Second, CT measurements were obtained in the supine position and therefore systematically underestimate upright deformity measures, as confirmed in our cohort (Cobb_CT 50.7° vs. Cobb_XR 54.6°; TK_CT 16.0° vs. TK_XR 24.6°). From a different perspective, however, persistence of substantial axial rotation in the supine position may itself serve as a surrogate of structural rotational rigidity, which may be more closely linked to ventilatory restriction than posture-dependent rotation. Third, we did not directly quantify rib cage geometry, thoracic volume, or lung volumes using three-dimensional segmentation, which would enable a more direct linkage between structural distortion and ventilatory limitation. Fourth, although both CT and spirometry were performed within a one-year interval, the precise per-patient interval was not systematically recorded, and interval changes in deformity or respiratory status—particularly in pediatric and adolescent patients undergoing growth—may have introduced additional variability that could bias the observed associations in either direction. Fifth, the cohort encompassed multiple etiologic categories (idiopathic, congenital, neuromuscular, and syndromic), each with distinct mechanisms of pulmonary impairment; pooled analyses may therefore reflect a composite of structural and non-structural contributions to ventilatory restriction. Although subgroup analyses were performed to address this heterogeneity, several subgroups had relatively small sample sizes (e.g., NF *n* = 18, SMA *n* = 20), and the resulting correlation estimates—including the strong correlation observed in NF (r = −0.87) and the weak correlation in SMA (r = −0.36)—should be interpreted with caution, as small samples may yield unstable estimates and inflated effect sizes at the extremes. Sixth, although Cobb angle and thoracic kyphosis were incorporated in sensitivity analyses, additional aspects of curve morphology—including apex location, curve type (e.g., Lenke classification [17]), and curve flexibility on bending films—were not modeled and may contribute additional explanatory information. Seventh, thoracic kyphosis was analyzed as a continuous variable, but its known non-linear (U-shaped) relationship with pulmonary function, in which both hypokyphosis and hyperkyphosis reduce lung volumes through distinct mechanisms, may have led to underestimation of its association in linear regression; future studies incorporating categorical or piecewise modeling are warranted. Finally, the observational, cross-sectional design permits identification of associations but does not establish causation; the consistency of associations across multiple spirometric endpoints and etiologic subgroups, however, supports the clinical relevance of global rotational asymmetry as a functional correlate.

### 4.2. Future Directions

Future research should expand on the present findings in several directions. First, the integration of machine learning and deep learning pipelines for fully automated vertebral segmentation and rotation measurement [18] could enable scalable, reproducible deployment of these indices in routine clinical workflows and reduce inter-observer variability inherent in manual measurement. Second, multicenter prospective studies are needed to externally validate the predictive value of ΔR(M–C) across diverse imaging platforms, scanner protocols, and ethnically heterogeneous populations. Third, longitudinal pediatric and adolescent cohorts should be followed serially to determine whether progression of rotational asymmetry tracks with deterioration in pulmonary function during the growth spurt, and whether early identification of patients with disproportionate ΔR(M–C) could refine timing of surgical intervention. Fourth, postoperative studies evaluating whether surgical correction of rotational asymmetry translates into measurable recovery of FVC and FEV1 would clarify the causal contribution of torsional deformity to ventilatory restriction. Finally, the complementary use of standing biplanar imaging (e.g., EOS), dynamic or four-dimensional CT, and rib cage and lung volume segmentation could connect rotation indices directly to thoracic volume restriction and chest wall mechanics, advancing the field toward a fully three-dimensional, function-oriented assessment of spinal deformity.

## 5. Conclusions

CT-derived vertebral rotation indices are significantly associated with pulmonary function impairment in scoliosis. Among evaluated indices, rotational asymmetry between the main and compensatory curves, ΔR(M–C), demonstrates the strongest explanatory performance for restrictive spirometric outcomes, outperforming apical rotation measures. Incorporating CT-based rotational asymmetry may enhance functional risk stratification and complement traditional radiographic assessment.

Global rotational asymmetry quantified by CT appears to capture a dimension of scoliosis deformity of potential biomechanical and functional relevance, offering a step toward three-dimensional, function-oriented assessment of spinal deformity.

## Figures and Tables

**Table 1:** Correlation between rotational asymmetry (ΔR(M–C)) and pulmonary function parameters by diagnostic subgroup.

Diagnostic Subgroup	*n*	FVC (L) (r, *p*)	FVC% (r, *p*)	FEV1 (L) (r, *p*)	FEV1% (r, *p*)
AIS	57	−0.60 (<0.001)	−0.52 (<0.001)	−0.54 (<0.001)	−0.46 (<0.001)
CMD	22	−0.55 (0.008)	−0.61 (0.002)	−0.54 (0.009)	−0.60 (0.003)
Congenital scoliosis	24	−0.44 (0.031)	−0.71 (<0.001)	−0.45 (0.028)	−0.61 (0.002)
DMD	34	−0.32 (0.066)	−0.41 (0.015)	−0.28 (0.111)	−0.34 (0.051)
SMA	20	−0.30 (0.198)	−0.36 (0.115)	−0.29 (0.217)	−0.41 (0.074)
Other neuromuscular	30	−0.38 (0.040)	−0.46 (0.011)	−0.39 (0.032)	−0.48 (0.008)
NF	18	−0.67 (0.002)	−0.87 (<0.001)	−0.65 (0.004)	−0.83 (<0.001)
Other syndromic	45	−0.38 (0.009)	−0.40 (0.007)	−0.40 (0.007)	−0.41 (0.006)

## Data Availability

The data presented in this study are available from the corresponding author upon reasonable request. The data are not publicly available due to privacy and ethical restrictions.

## Supplementary Materials

The following supporting information can be downloaded at: https://www.mdpi.com/article/10.3390/jcm15114154/s1, Supplementary Table S1: Multivariate regression coefficients for rotation indices predicting pulmonary function. Supplementary Table S2: Variance inflation factors (VIFs) for multivariate models. Supplementary Table S3: Incremental adjusted R^2^ (Δadjusted R^2^) for rotation indices beyond covariates-only models.

## References

[1] Aaro S., Dahlborn M. (1981). Estimation of vertebral rotation and the spinal and rib cage deformity in scoliosis by computer tomography. Spine.

[2] Stokes I.A.F. (1994). Three-dimensional terminology of spinal deformity. Spine.

[3] Weinstein S.L., Dolan L.A., Cheng J.C.Y., Danielsson A., Morcuende J.A. (2008). Adolescent idiopathic scoliosis. Lancet.

[4] Koumbourlis A.C. (2006). Scoliosis and the respiratory system. Paediatr. Respir. Rev..

[5] Harris J.A., Mayer O.H., Shah S.A., Campbell R.M., Balasubramanian S. (2014). A comprehensive review of thoracic deformity parameters in scoliosis. Eur. Spine J..

[6] Newton P.O., Faro F.D., Gollogly S., Betz R.R., Lenke L.G., Lowe T.G. (2005). Results of preoperative pulmonary function testing of adolescents with idiopathic scoliosis. J. Bone Jt. Surg. Am..

[7] Kearon C., Viviani G.R., Kirkley A., Killian K.J. (1993). Factors determining pulmonary function in adolescent idiopathic thoracic scoliosis. Am. Rev. Respir. Dis..

[8] Johari J., Sharifudin M.A., Ab Rahman A., Omar A.S., Abdullah A.T., Nor S., Lam W.C., Yusof M.I. (2016). Relationship between pulmonary function and degree of spinal deformity, location of apical vertebrae and age among adolescent idiopathic scoliosis patients. Singap. Med. J..

[9] Skalli W., Lavaste F., Descrimes J.L. (1995). Quantification of three-dimensional vertebral rotations in scoliosis: What are the true values?. Spine.

[10] Wang Y., Yang F., Wang D., Zhao H., Ma Z., Ma P., Hu X., Wang S., Kang X., Gao B. (2019). Correlation analysis between the pulmonary function test and the radiological parameters of the main right thoracic curve in adolescent idiopathic scoliosis. J. Orthop. Surg. Res..

[11] Farrell J., Garrido E. (2021). Predicting preoperative pulmonary function in patients with thoracic adolescent idiopathic scoliosis from spinal and thoracic radiographic parameters. Eur. Spine J..

[12] Ohashi M., Watanabe K., Hirano T., Hasegawa K., Katsumi K., Shoji H., Mizouchi T., Endo N. (2020). Flexibility of the thoracic curve and three-dimensional thoracic kyphosis can predict pulmonary function in nonoperatively treated adult patients with adolescent idiopathic scoliosis. J. Orthop. Sci..

[13] Graham B.L., Steenbruggen I., Miller M.R., Barjaktarevic I.Z., Cooper B.G., Hall G.L., Hallstrand T.S., Kaminsky D.A., McCarthy K., McCormack M.C. (2019). Standardization of spirometry 2019 update. An official American Thoracic Society and European Respiratory Society technical statement. Am. J. Respir. Crit. Care Med..

[14] Quanjer P.H., Stanojevic S., Cole T.J., Baur X., Hall G.L., Culver B.H., Enright P.L., Hankinson J.L., Ip M.S.M., Zheng J. (2012). Multi-ethnic reference values for spirometry for the 3–95-yr age range: The global lung function 2012 equations. Eur. Respir. J..

[15] Cobb J.R. (1948). Outline for the study of scoliosis. American Academy of Orthopaedic Surgeons; Instructional Course Lectures.

[16] Farrell J., Garrido E. (2018). Effect of idiopathic thoracic scoliosis on the tracheobronchial tree. BMJ Open Respir. Res..

[17] Lenke L.G., Betz R.R., Harms J., Bridwell K.H., Clements D.H., Lowe T.G., Blanke K. (2001). Adolescent idiopathic scoliosis: A new classification to determine extent of spinal arthrodesis. J. Bone Jt. Surg. Am..

[18] Adam C.J., Askin G.N. (2006). Automatic measurement of vertebral rotation in idiopathic scoliosis. Spine.

