# Supplementary material for: A Quantitative CT-Based Analysis of Vertebral Rotational Asymmetry and Pulmonary Function in Scoliosis
Source: J Clin Med. 2026 May 28;15(11):4154. doi: 10.3390/jcm15114154 (PMC13258114; doi:10.3390/jcm15114154)
Supplement: Supplementary file 1 [file jcm-15-04154-s001.zip › jcm-4299848-supplementary.pdf]

Supplementary Material

Supplementary Table S1. Multivariate regression coefficients for rotation indices predicting pulmonary function.

Outcome	Predictor	β	SE	p-value
FVC%	R(Apex)	−0.46	0.05	<0.001
FVC%	R(Avg)	−0.47	0.05	<0.001
FVC%	ΔR(M–C)	−0.58	0.05	<0.001
FEV1%	R(Apex)	−0.45	0.05	<0.001
FEV1%	R(Avg)	−0.46	0.05	<0.001
FEV1%	ΔR(M–C)	−0.57	0.05	<0.001

β indicates standardized regression coefficient.

Supplementary Table S2. Variance inflation factors (VIFs) for multivariate models.

Variable	VIF
Age	1.09
Sex	1.02
Height	1.94
Weight	2.00
R(Apex)	1.06
R(Avg)	1.07
ΔR(M–C)	1.12

All VIF values were < 5, indicating no significant multicollinearity. VIFs were computed in separate models including covariates and one rotation index at a time.

Supplementary Table S3. Incremental adjusted R^2 (Δadjusted R^2) for rotation indices beyond covariates-only models.

Outcome	n	Covariates only	+R(Apex)	ΔAdj R^2	+R(Avg)	ΔAdj R^2	+ΔR(M–C)	ΔAdj R^2
FEV1 (L)	250	0.50	0.59	0.09	0.60	0.10	0.65	0.15
FEV1%	250	0.18	0.37	0.19	0.38	0.20	0.48	0.30
FVC (L)	250	0.51	0.60	0.10	0.61	0.10	0.66	0.16
FVC%	250	0.20	0.40	0.20	0.41	0.21	0.51	0.31
